# Severe Diabetic Gastroparesis and Complex Discharge Planning: Home IV Cyclizine As the Only Solution

**DOI:** 10.7759/cureus.100805

**Published:** 2026-01-05

**Authors:** Hera Karim, Aysel Ibrahimli, Taiwo Ikuesan

**Affiliations:** 1 General Medicine, King's College Hospital NHS Foundation Trust, London, GBR; 2 Internal Medicine, King's College Hospital NHS Foundation Trust, London, GBR

**Keywords:** complex discharge planning, diabetic gastroparesis (dg), diabetic ketoacidosis (dka), g-poem, iv cyclizine, mdt collaboration, nephrotic syndrome

## Abstract

Gastroparesis is a chronic disorder characterised by delayed gastric emptying in the absence of a physical blockage. Gastroparesis can be clinically classified as mild (Grade 1), moderate (Grade 2), and severe (Grade 3), based on the severity of symptoms, with grade 3 being refractory or intractable symptoms that are not controlled despite medical therapy. Gastroparesis can occur in both type 1 and type 2 diabetes; it is more common in type 1 diabetes. Severe gastroparesis in type 1 diabetes mellitus (T1DM) can be very difficult to manage, especially when accompanied by multi-organ complications. This case highlights the importance of individualised discharge planning for a young adult with severe diabetic gastroparesis, where long hospital stays, difficult-to-treat symptoms, and multiple health problems make it very challenging to achieve quality of life and independence outside the hospital.

A 29-year-old patient was admitted to the hospital with severe vomiting due to T1DM-related gastroparesis. Her case was complicated by other comorbidities, such as chronic kidney damage and nephrotic syndrome, persistent hypertension, electrolyte disturbances, and frequent diabetic ketoacidosis. During her long hospital admissions, she was started on multiple antiemetic and prokinetic medications as well as undergoing an invasive procedure of G-POEM (gastric peroral endoscopic myotomy) with no success in symptom management. No improvement was appreciated in gastric emptying studies. Due to difficulty in symptom management and dependency on IV cyclizine, which was the only medication relieving her symptoms, combined with her complex health problems and medical needs, she was compelled to stay in hospital, causing a 14-months-long hospital stay on one occasion, followed by multiple admissions, all due to same reason and a final 4-months stay when multiple multidisciplinary team (MDT) meetings were done to help her live her day-to-day life without being isolated in the hospital and give her a level of autonomy by being discharged on IV cyclizine.

This case highlights the importance of an individualised patient care plan when the conventional methods fail to achieve the necessary outcome for young patient’s general wellbeing and control over her life without being affected by uncontrolled symptoms, as well as the importance of MDT collaboration, when all the different healthcare professionals come together to ensure the safety of IV home medication is supported by governance and community nursing support.

## Introduction

Gastroparesis is a chronic disorder in which the stomach empties food more slowly than normal, without any physical blockage being present [1]. It presents with nausea, vomiting, early satiety, bloating, abdominal pain, and poor oral intake, leading to nutritional deficiencies and impaired quality of life [2]. Its pathophysiology is multifactorial; many patients with type 1 diabetes mellitus (T1DM) suffer from gastroparesis due to autonomic neuropathy as well as abnormalities in gastric muscle and pacemaker function [3].

The prevalence of gastroparesis varies among diabetics, but studies show that the incidence of diabetic gastroparesis has been estimated to be 5.2% in patients with type 1 diabetes. The prevalence of diabetes-associated gastrointestinal symptoms is 5-12% [3,4].

Severe cases often present with unstable blood sugars, electrolyte problems, repeated hospital stays, and episodes of diabetic ketoacidosis (DKA)[5,6]. Beyond physical symptoms, gastroparesis also has a big impact on mental health and can affect one's independence significantly [7,8].

Management can often be very challenging. Usually, management options for gastroparesis start with dietary changes, followed by prokinetic medications and antiemetics; however, these do not always provide patients with adequate relief, and outcomes can be short-term [9]. When symptom control is not achievable with those options, there are also invasive procedures available, such as G-POEM (gastric per-oral endoscopic myotomy), to give patients the required symptom control, but the short-term and long-term outcomes can vary from patient to patient [10, 11]. For G-POEM, the technical success rate is 100%, and the short-term (within 1 year) success rate is about 50-80%. [12].

This case illustrates the management challenges in a young patient with T1DM, which was complicated by severe gastroparesis and multi-organ comorbidities. It shows how standard treatment options can be limited and why multiple specialists need to work together in decision-making in order to provide the best possible outcome for patients. Additionally, it shows us the importance of shaping care around patient needs by describing how multiple treatment options, including invasive interventions, failed, yet with the help of multidisciplinary teams (MDTs), the patient was able to be discharged on IV cyclizine - the only promising therapeutic option proven for the patient.

## Case presentation

A 29-year-old female presented with recurrent, non-bloody, and non-projectile vomiting for a couple of months on a known background of type 1 diabetes mellitus. She had only just been discharged home a few days prior to this admission. This was her tenth admission for the same complaints during the previous 1-year period. Vomiting was described by her as “too many times to count”. Investigations showed ketosis with significant acidosis. The diabetic team continued to review her to provide guidance on her management and suggested adjustments to her diabetic medications.

**Figure 1:**
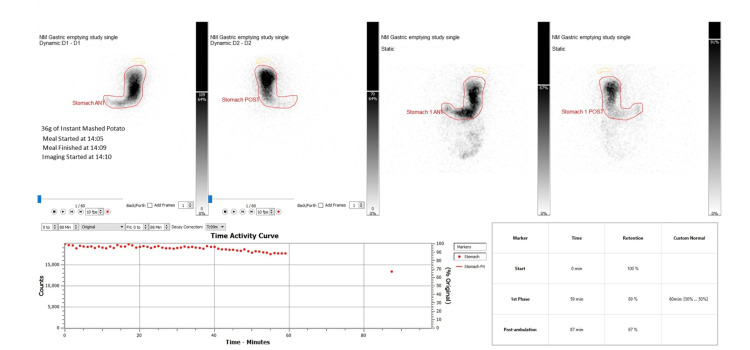
Gastric emptying studies pre-G-POEM IN 2023 G-POEM: Gastric Peroral Endoscopic Myotomy

Gastric emptying studies were done, which showed - 59 min retention of 89%, 87 min retention of 67% -that confirmed delayed gastric emptying (Figure 1). She was treated with multiple antiemetics, but despite being on three different antiemetics at a time, including cyclizine, ondansetron, and metoclopramide, her vomiting was consistent. Later, a trial of domperidone was also tried with no success, and due to the risk of prolongation of QTc, it was stopped after a few days. OGD was performed to investigate any mechanical cause of vomiting; the results showed nothing significant, but oesophageal candidiasis that was treated with anti-fungal.

The gastroenterology team then decided to do the Gastric Peroral Endoscopic Myotomy (G-POEM) following optimisation of her glycaemic control. Meanwhile, she was treated with multiple other antiemetics, including aprepitant, prucalopride, and erythromycin for intractable gastroparesis while awaiting G-POEM, which also failed to relieve her symptoms, and she continued to have multiple diabetic ketoacidosis episodes and electrolyte imbalances requiring diabetes, renal, and dietitian inputs. G-POEM was done, which was another failure, as it comes with a short response of a few days, then the symptoms returned.

OGD with Botox injection to the pylorus was performed with the aim of relieving her distressing symptoms, but her symptoms worsened following the Botox injection. An MRI head was even done to rule out brain pathology, but it also came back negative.

Subsequent to multiple failed endeavours, a gastric pacemaker was also suggested by the MDT, but as the NHS no longer routinely provides this service, the idea was dismissed.

After numerous unsuccessful attempts, it was suggested by the gastroenterology team to try neuromodulators such as imipramine and amitriptyline; however, it had a negligible impact on the symptoms. In addition to that, promethazine and azithromycin one by one were also administered as a trial but stopped later as they provided limited advantage to her.

Cyclizine and domperidone were not suitable as long-term treatment options because of the element of dependence on cyclizine and the risk of arrhythmias due to domperidone; the latter was stopped. The medical team tried to wean her off IV cyclizine multiple times due to the element of dependence on it, but she kept relapsing and said that was the only medicine that worked for her. It was even tried switching to oral and IM routes, but it was not as effective as IV cyclizine, so it was switched back to the IV route. Due to her continuing sickness and IV cyclizine dependency, a specialist addiction psychiatry evaluation was conducted, and as no addiction behaviour was identified, it was continued (Table 1).

**Table 1:** Summary of Pharmacological Treatments Trialled During Admission This table summarises the pharmacological therapies trialled during a prolonged inpatient admission for severe gastroparesis. Medications were introduced sequentially or re-trialled intermittently based on clinical response and tolerability. Duration reflects cumulative exposure where applicable. Intravenous cyclizine was administered throughout admission and later delivered via a syringe driver to facilitate discharge planning. Discontinuation reasons include lack of efficacy and safety concerns, such as QT interval prolongation.

Medication	Dose and Route	Frequency	Duration	Outcome
Cyclizine	50 mg IV	TDS PRN	Throughout admission	Only treatment providing sustained symptom control. Multiple attempts to switch to oral therapy or reduce dosing frequency were unsuccessful. For discharge planning, IV cyclizine was changed to a syringe driver on discharge following 13 months of hospital admission.
Metoclopramide	10 mg PO	TDS (regular and PRN)	2 weeks regular followed by PRN	No clinical benefit; discontinued due to QT prolongation risk.
Ondansetron	4 mg PO	BD/TDS PRN	2 weeks regular followed by PRN	No improvement in symptoms
Domperidone	10 mg PO	TDS (regular and PRN)	Re-trialled on multiple occasions with a cumulative duration of 13 months	Trialled on multiple occasions during admission; discontinued due to QT prolongation risk. Discharged after 13 months hospital admission
Aprepitant	80 mg PO	OD	28 weeks in total	No significant symptom improvement, but restarted on discharge following 13 months of hospital admission as per gastroenterology advice.
Erythromycin	250 mg PO and IV	TDS	10 weeks	No benefit despite oral and intravenous trials and dose increase (500 mg).
Haloperidol	1–2 mg PO daily; 25 mg IM once monthly	OD/single dose	4 weeks, IM single dose was only administered once	Limited benefit; discontinued due to QT prolongation risk.
Prucalopride	2 mg PO	OD	44 weeks	No significant improvement. Discharged on it after 13 months of hospital admission.
Imipramine	10 mg PO	BD	13 weeks	Discontinued following gastroenterology team advice, was replaced by amitriptyline.
Amitriptyline	10 mg PO	OD	7 weeks	Discontinued due to lack of efficacy by gastroenterology team.
Prochlorperazine	5 mg PO	TDS	4 weeks	No clinical improvement.
Azithromycin	250 mg, increased to 500 mg PO	OD	4 weeks in total	No benefit as a prokinetic agent.
Amisulpride	50 mg PO	OD	40 weeks	No clear clinical benefit. Discharged on it after 13 months of hospital admission.
Botulinum toxin	100 units (endoscopic intrapyloric injection)	Single procedure	N/A	No sustained symptomatic improvement.

**Figure 2:**
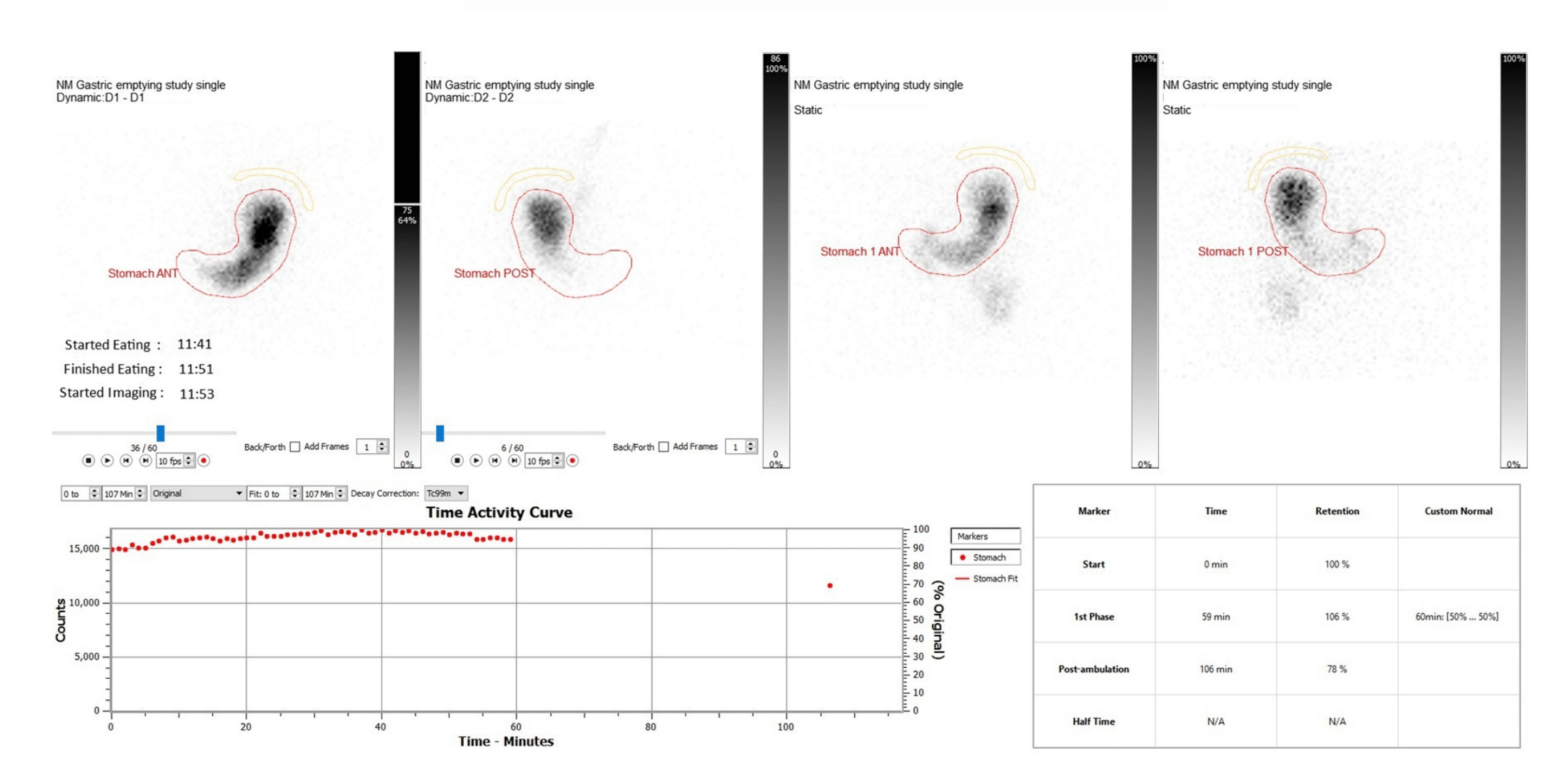
Gastric emptying studies post-G-POEM in 2024 G-POEM: Gastric Peroral Endoscopic Myotomy

Repeat gastric emptying study was carried out after 7 months, which demonstrated no improvement post procedure and persisting severe delayed gastric emptying study, results showed - 59 min retention of 106%, 106 min retention of 78% (Figure 2). At this time, she was still requiring IV cyclizine once daily and up to three doses on sick days.

Gastric emptying scintigraphy (GES) is the gold standard test for diagnosing gastroparesis. It measures how quickly a radiolabelled solid or liquid meal leaves the stomach [13]. Severity is assessed using gastric retention at 4 hours after the meal: less than 15% indicates mild disease, 15-35% moderate disease, and more than 35% severe gastroparesis [13].

Multiple professional meetings were conducted in the interim, including gastroenterology, diabetes, and psychology teams, to consider if NJ feeding can give a long-term strategy, as stomach bypass would allow medications to be delivered distally and for her to leave the hospital, but she refused this procedure as she thought it would not help her symptoms, and her lifestyle would be affected.

As another possibility, she was treated with oral haloperidol with a plan that if she tolerated haloperidol, then to discontinue cyclizine, but it contributed very little, and eventually haloperidol had to be stopped.

OGD with endoflip was carried out, the results of which were consistent with delayed gastric emptying as the pylorus was widely opened and there was no indication to repeat G-POEM.

Gastric electromyography (EMG) was also suggested by the gastro team; however, she declined this.

Following numerous failed strategies, it was suggested by the MDT that she should be treated with amisulpride, for which an individual funding request was submitted, upon approval of which it was started.

Being in hospital for more than a year, it was decided to alter the cyclizine regimen to facilitate her discharge, i.e., one dose oral and the other doses by IV route, making no difference. Finally, the syringe pump was started as a measure for weaning of IV cyclizine, on which she appeared to be increasingly dependent in anticipation of discharge.

**Figure 3:**
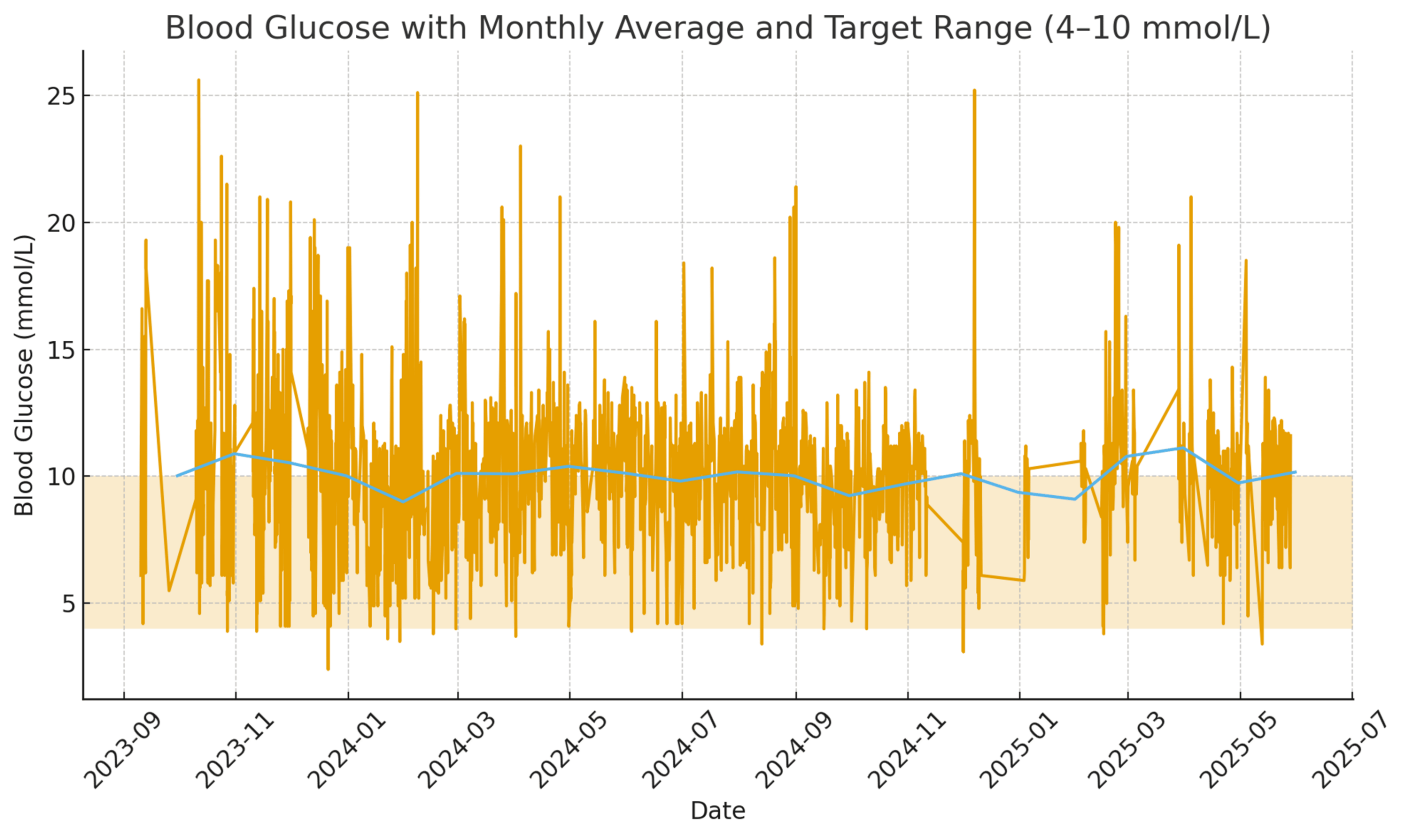
Daily blood glucose readings over a period from September 2023 to June 2025 The orange line shows day-to-day glucose readings. The blue line shows the monthly average, which represents the overall glycaemic trend. The yellow shaded band is the ideal glucose target range, 4-10 mmol/L.

**Figure 4:**
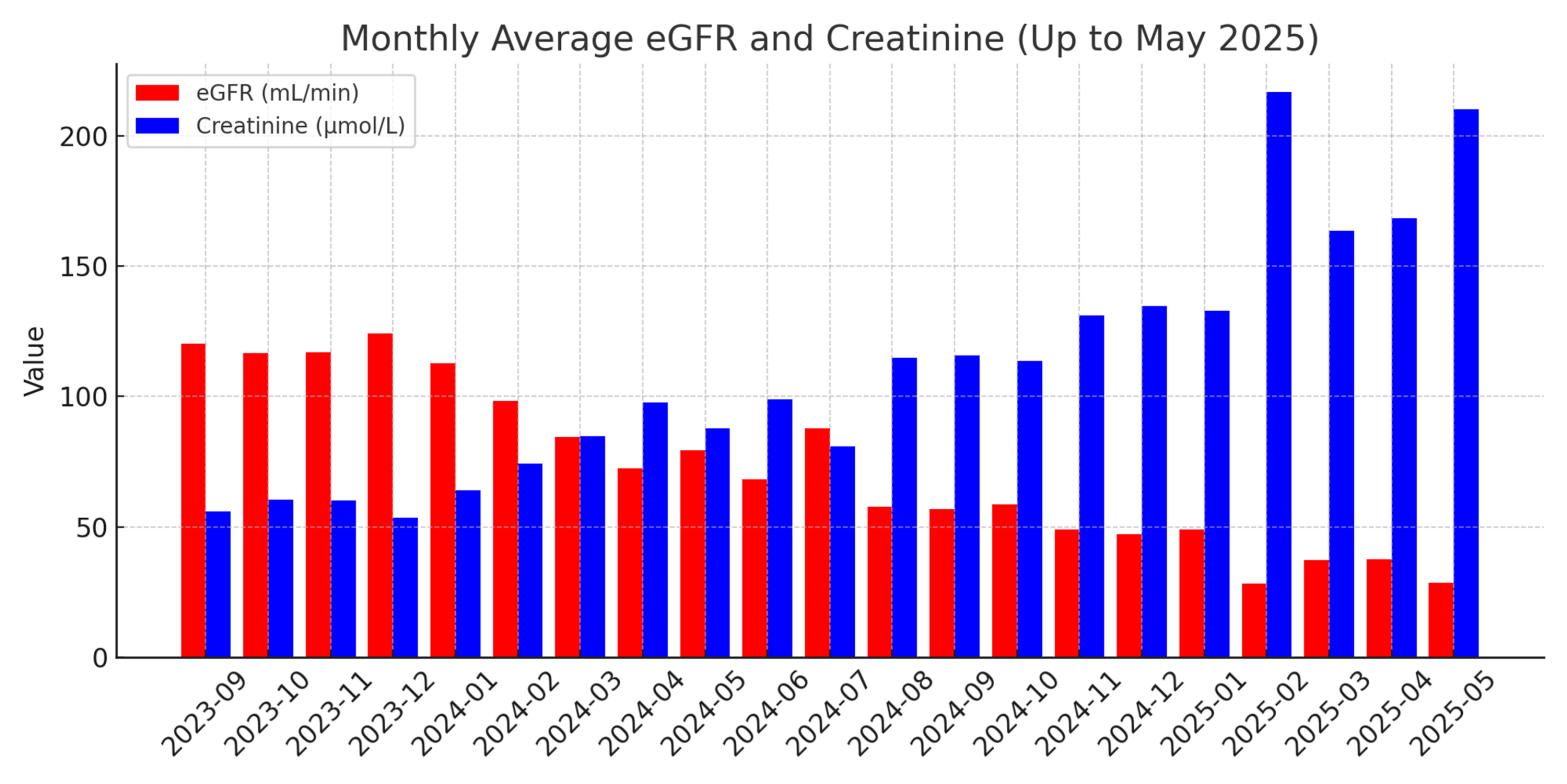
Monthly average eGFR and creatinine levels from September 2023 to May 2025. Red bars represent the monthly average eGFR readings (normal range for eGFR>90 mL/min/1.73 m²). Blue bars represent the monthly average Creatinine values (normal range for creatinine values 45 to 84 µmol/L).

The patient’s blood glucose levels remained poorly controlled during the admission, this marked by day-to-day variability and frequent spikes above the target glucose range (Figure 3). In addition, her renal function progressively deteriorated over time, demonstrated by falling eGFR and rising creatinine levels (Figure 4).

The other significant events that happened during this long admission were recurrent DKA episodes, acute kidney injury, and electrolyte imbalance, including significant hyperkalaemia due to recurrent vomiting leading to dehydration, multiple failed venous access requiring Hickman line insertion, low mood necessitating psychiatric input, and hypothalamic amenorrhea secondary to domperidone-induced hyperprolactinemia, calling for endocrine input.

After a 14-month hospital stay, she was finally able to be discharged on a syringe driver with hospital-at-home team input.

After this protracted admission, she continued to have further multiple admissions for the same symptoms, including another long admission (4-month stay) because the cyclizine pump was not an effective relief for her symptoms. It was discussed with the hospital-at-home team and concluded to support the idea of switching her to IV cyclizine with consideration of training her to self-administer in the community. While inpatient, she began training under the teaching of nurses to self-administer cyclizine; however, the training was abandoned after a governance and safety issue was raised. As a result of this, extensive multi-professional meetings were held in regards to her discharge plan given the complexity of the management of her symptoms and medical background, and eventually it came to the unanimous conclusion agreed upon by the medical, endocrine and gastroenterology consultants, hospital-at-home team lead, inpatient nursing team and district nurses that in this exceptional case, she would be trained to self-administer IV cyclizine in the community with satisfactory training competency. Hence, she was finally discharged on IV cyclizine, making it an exceptionally complex and challenging discharge.

## Discussion

Refractory gastroparesis is difficult to manage and often requires escalation to invasive treatments such as jejunal feeding tubes, gastric electrical stimulation, or endoscopic procedures. Despite these approaches, some patients remain severely symptomatic with repeated hospital admissions. Reports of long-term intravenous antiemetic therapy in the community are very limited, highlighting the rarity and clinical complexity of this case.

This young patient had trials of many antiemetics, prokinetics, and even G-POEM, but despite all these, her symptoms continued. Gastric emptying studies showed no improvement in delayed emptying, which caused the symptoms. Her longest hospital admission lasted over 13 months, followed by multiple more admissions, including a final four-month stay. Such long hospital stays are not common, but have been reported in severe and treatment-resistant cases [7]. The only medication that provided consistent symptom control was IV cyclizine.

IV cyclizine is commonly used in hospitals; however, there is little evidence to support its long-term use in gastroparesis. Additionally, there are safety concerns regarding the use of intravenous medications outside the hospital setting, including infection related to long-term IV access and potential medication side effects, which have limited wider adoption of this approach [8]. In this case, these risks were managed through a structured, MDT-led governance framework. This included clear prescribing responsibility, competency-based IV and line-care training with initial supervision in hospital, and ongoing support from district nursing teams following discharge. IV cyclizine was supplied in limited quantities with regular clinical review. A clear escalation plan was agreed upon, with medical review required before any IV fluids were given due to the risk of fluid overload.

In this patient's situation, the risk of keeping her in the hospital for the long term was decided to have more risks than providing her with IV cyclizine at home with strong governance and hospital-at-home team support.

The impact on this young female’s life was significant, considering she had to spend months in the hospital and had no quality of life outside of the hospital ward. This reflects what other studies show in terms of the impact gastroparesis can have; it not only causes physical symptoms, but also affects mental health, resulting in disruptions to daily life, work, and other aspects of life [7,8].

Thanks to MDT collaboration and a holistic approach created by different specialists, she gained her independence and ability to live comfortably without needing to be admitted to the hospital. This case underlines how limited current options are for patients with severe gastroparesis and how important it is to work as a team. It also shows that sometimes, safe and innovative solutions - like home IV antiemetics under strong governance may be needed to reduce hospital stays and give patients back some independence.

## Conclusions

This case illustrates the importance of involvement of MDT, especially in complex cases when standard treatment fails; care must be adapted to the patient’s needs. Although this approach was unusual and depended on specialised resources, trained staff, and close monitoring, it was made possible through strong teamwork. With careful planning and clear safety measures, the team was able to safely deliver IV cyclizine at home, which helped manage one’s symptoms, reduced hospital admissions, and gave the young patient more independence. This intervention not only addressed one's immediate health concerns but also allowed for a more sustainable, patient-centred approach to care. Ultimately, an individualised discharge plan can improve a patient’s quality of life when other treatments are no longer effective. Although long-term outcomes are uncertain and risks remain, this case suggests that home IV antiemetics may be considered in carefully selected patients with severe gastroparesis under strict MDT governance, rather than as a broadly generalisable approach.
